# Increased plasma levels of NT-proBNP, Troponin T and GDF-15 are driven by persistent AF and associated comorbidities: Data from the AF-RISK study

**DOI:** 10.1016/j.ijcha.2022.100987

**Published:** 2022-03-08

**Authors:** L.M.G. Meems, V. Artola Arita, M. Velt, E.A.M.P. Dudink, H.J.G.M. Crijns, I.C. Van Gelder, M. Rienstra

**Affiliations:** a Department of Cardiology, University Medical Center Groningen, University of Groningen, Groningen, the Netherlands; b Maastricht University Medical Center+ and Cardiovascular Research Institute Maastricht, Maastricht, the Netherlands

**Keywords:** Atrial fibrillation, Biomarkers, NTproBNP, Growth-differentiation factor 15, Troponin T, AF, atrial fibrillation, HF, heart failure, pAF, paroxysmal atrial fibrillation, NTproBNP, N-terminal pro-brain natriuretic peptide, GDF-15, growth differentiation factor-15, EHRA, European Heart Rhythm Association, LVEF, left ventricular ejection fraction, LAVI, left atrial volume indices

## Abstract

Atrial fibrillation (AF) is a progressive disease, and early recognition and management may reflect an important strategy to reduce its disease burden. In this study, we evaluated plasma levels of three biomarkers - N-terminal pro-brain natriuretic peptide (NTproBNP), Troponin-T, and growth differentiation factor-15 (GDF-15) - in patients with paroxysmal AF (pAF) (≤7 days of continuous AF, n = 323) and persistent AF ((AF duration > 7 days and < 1 year, n = 84) using patients from *AF RISK* study (NCT01510210). In this AF-RISK sub-study, patients with persistent AF experienced more symptoms (higher European Heart Rhythm Association class (p < 0.001)), had a higher comorbidity burden (p < 0.001), and had more unfavorable echocardiographic parameters (p < 0.001). All three biomarker levels were significantly higher in patients with persistent AF as compared to those with pAF (p < 0.001). Multivariate linear regression analyses showed that age (beta-coefficient for NTproBNP: 0.21; GDF-15: 0.41; Troponin-T: 0.23) and CHA_2_DS_2_-VASc (beta-coefficient for NTproBNP: 0.20; GDF-15: 0.25; Troponin-T: 0.27) were determinants of all three biomarkers, and that persistent AF determined NTproBNP (beta-coefficient: 0.34), but not Troponin-T and GDF-15. More detailed analysis of CHA_2_DS_2_-VASc score showed that for all three biomarkers age, coronary artery disease and heart failure were determinants of plasma biomarkers levels, whereas sex determined NTproBNP and Troponin T, and hypertension determined NTproBNP and GDF15. Overall, this study therefore suggests that in AF, Troponin T and GDF15, and especially NTproBNP could be used to detect those patients with more persistent form of AF that may warrant more aggressive treatment of AF and concomitant comorbidities. Future studies, however, are essential to evaluate if more aggressive AF treatment and risk factor management will reduce disease progression and holds a novel therapeutic intervention to reduce the burden of AF.

Atrial fibrillation (AF) is the most common cardiac arrhythmia, with still increasing incidence and prevalence rate. It is expected that 6–12 million people will suffer this condition in 2050 in the US, and 17.9 million people in Europe by 2060 [1]. Patients with AF are known to have increased mortality and morbidity rates, including the development of dementia, heart failure (HF) and stroke [2], [3], and this causes an important economic and health care burden. AF is a progressive disease, and early recognition and management of increasing AF burden may reflect an important strategy to reduce its disease burden [4].

In this study, we therefore evaluated plasma levels of three biomarkers in patients with paroxysmal AF (pAF) and persistent AF (persistent AF) using patients from *Identification of a risk profile to guide atrial fibrillation therapy (AF RISK)* study (NCT01510210). Detailed study design and outcomes have been previously described [5]. In short, AF RISK was a multicenter, prospective, observational study, including patients aged ≥ 18 years, with pAF (total AF history < 2 years, or total AF history < 3 years in case of ≤ 2 AF episodes of ≤ 48 h per month terminating spontaneously) or persistent AF (total AF history < 2 years, and total persistent AF duration > 7 days and < 1 year) in whom a rhythm control strategy was preferred. AF RISK was performed in compliance of the Declaration of Helsinki. The institutional review board approved study protocol and all patients gave written informed consent. After inclusion, all patients underwent baseline assessment including peripheral venous blood sampling for biomarker analyses. Blood samples were processed and EDTA-plasma samples were stored at −80C. For present analysis, we included pAF patients who had sinus rhythm during blood sampling (n = 323) and included persistent AF patients who were known with persistent AF, but were in sinus rhythm at baseline visit, either by scheduled cardioversion or spontaneous conversion, and had atrial fibrillation during blood sampling (n = 84). Patients with pAF and AF during blood sampling were excluded from this sub-study (n = 8).

We analyzed plasma levels of two traditional cardiovascular biomarkers - N-terminal pro-brain natriuretic peptide (NTproBNP) and Troponin-T – and one non-traditional cardiovascular biomarker -growth differentiation factor-15 (GDF-15).Troponin-T, NTproBNP and GDF-15 were measured at baseline using electrochemiluminescence by a Cobase 411 analyser using a standard Roche Diagnostics GmbH method.

Descriptive data of continuous variables are presented as mean ± standard deviation in normally distributed data or median (interquartile range) in non-normally distributed data. Categorical variables are presented as numbers with percentages. Differences between groups were evaluated by Student’s *t*-test or Mann–Whitney *U* test for continuous variables, and Chi-square test was used for categorical variables. A p-value < 0.05 was considered statistically significant. Linear regression analyses were performed to determine risk indicators of NTproBNP, GDF15 and Troponin T levels. Biomarkers were logarithmically transformed and standardized to realize constant variance. All patient characteristics and biomarkers were tested using a stepwise approach: univariate variables with p < 0.1 were investigated in a multivariate model. In the multivariate model, a variable was excluded when p ≥ 0.05. Since CHA_2_DS_2_-VASc is a surrogate parameter that is a composite score of several components (sex, age, hypertension, diabetes mellitus, coronary artery disease, HF, peripheral artery disease, thromboembolic events) association between three different biomarkers and individual components were tested separately for each biomarker using linear regression analysis. Statistical analyses were performed using R package (Version 3.1.3; R Foundation for Statistical Computing, Vienna, Austria) or STATA (Version 14.2; StataCorp LLC; Tx, USA).

In this AF-RISK sub-study, patients with pAF were younger, more often women and had lower CHA_2_DS_2_-VASc scores (Table 1). Patients with persistent AF experienced more symptoms as reflected by higher European Heart Rhythm Association (EHRA) class (p < 0.001). They also had mildly impaired kidney function and suffered from more comorbidities, including hypertension, congestive heart failure and a history of coronary artery disease (Table 1). Transthoracic echocardiography showed higher left ventricular ejection fraction (LVEF) in patients with pAF (LVEF %, median [IQR]: 58 [58–60]) and smaller left atrial volume indices (LAVI ml/m2, median [IQR]: 32 [25–39]) as compared to those patients with persistent AF (LVEF %, median [IQR]: 55 [50–58], p < 0.001; LAVI ml/m2, median [IQR]: 39 [31–47], p < 0.001).

**Table 1:** Baseline characteristics of patients with paroxysmal AF (pAF) and persistent AF. Results are presented as mean ± standard deviation; median [Interquartile ranges] in case of continuous data. For categorical data results are presented as percentages. P < 0.05 is considered significant. Differences in continuous data were tested by Student’s *t*-test or Mann-Whitney *U* test depending on normality of data. Differences in categorical data were tested by χ^2^-test. HF = heart failure; CAD = coronary artery disease; TIA = transient ischemic attack; CVA = cerebrovascular accident; EHRA = European heart rhythm association; eGFR = estimated glomerular filtration rate; LVEF = left ventricular ejection fraction; LVEDD = left ventricular end diastolic dimension; LAVI = left atrial volume index; ACEi = angiotensin converting enzyme inhibitor; ARB = angiotensin receptor blocker; Ca = calcium; AAD = anti arrhythmic drug; OAC = oral anticoagulant; NOAC = novel oral anticoagulant.

	Paroxysmal AF (n = 323)	Persistent AF (n = 84)	p-value
Age (years) (SD)	58 ± 12	63 ± 9	P < 0.001
Female sex (%)	130 (40)	19 (23)	P < 0.01
Blood pressure (mmHg)			
systolic	131 ± 18	129 ± 17	P = NS
diastolic	78 ± 9	80 ± 12	P = NS
Smoking (%)	41 (13)	17 (22)	P = NS
Diabetes Mellitus (%)	32 (9)	9 (11)	P = NS
Hypertension (%)	146 (45)	51 (61)	P < 0.05
Hypercholesterolemia (%)	121 (38)	35 (42)	P = NS
Chronic HF (%)	17 (5)	24 (29)	P < 0.001
CAD (%)	18 (6)	17 (20)	P < 0.001
TIA/CVA (%)	20 (6)	6 (7)	P = NS
EHRA class (%) I II III	101 (31) 177 (55) 44 (14)	19 (23) 46 (45) 27 (32)	P < 0.001
CHA_2_DS_2_VASc	1 [1–2]	2 [1–3]	P < 0.01
eGFR (ml/min/1,73 m2) LVEF (%)	86 [76–100] 58 [55–60]	75 [59–92) 55 [50–58]	P < 0.001 P < 0.001
LVEDD (mm)	49 [45–53]	50 [45–55]	P = NS
LAVI (ml/m2)	32 [25–39]	39 [31–47]	P < 0.001
Concomitant medication (%) Beta-blockers ACEi/ARB Ca-antagonists Digoxin Class IAAD Class IIIAAD (N)OAC Statin Diuretics	186 (58) 131 (40) 67 (21) 7 (2) 35 (11) 19 (6) 194 (60) 93 (29) 51 (16)	59 (70) 46 (56) 17 (20) 11 (13) 2 (2) 7 (8) 78 (93) 33 (39) 31 (37)	P < 0.05 P < 0.05 P = NS P < 0.001 P < 0.05 P = NS P < 0.001 P = NS P < 0.001
NTproBNP (pg/mL)	100 [46–228]	709 [321–1441]	P < 0.001
Troponin T (pg/mL)	5 [3–9]	8 [5–13]	P < 0.001
GDF15 (pg/mL)	882 [663–1245]	1152 [843–1647]	P < 0.001

All three biomarker levels were significantly higher in patients with persistent AF as compared to those with pAF: GDF-15, persistent AF: 1152 pg/mL [843–1647] versus pAF: 882 pg/mL [663–1245], p < 0.001; NTproBNP, persistent AF: 709 pg/mL [321–1441] versus pAF: 100 pg/mL [46–228], p < 0.001; Troponin T, persistent AF: 8 pg/mL [5–13] versus pAF: 5 pg/mL [3], [4], [5], [6], [7], [8], [9], p < 0.001 (Table 1). Multivariate linear regression analyses showed that age and CHA_2_DS_2_-VASc are determinants of all three biomarkers, but that other determinants were biomarker-specific. For example, NTproBNP is determined by age, CHA_2_DS_2_-VASc, increasing EHRA category, LAVI LVEF and presence of persistent AF; GDF15 is determined by age, CHA_2_DS_2_-VASc, smoking and LVEF; Troponin-T is determined by age, CHA_2_DS_2_-VASc and sex (Table 2). Since CHA_2_DS_2_-VASc is a composite score of several variables components (sex, age, hypertension, diabetes mellitus, coronary artery disease, HF, peripheral artery disease, pulmonary embolism or deep venous thrombosis, or cerebrovascular incident) we analyzed the effect of individual components per biomarker in more detail and observed that with regards to CHA_2_DS_2_-VASc score NTproBNP is determined by age, sex, hypertension, coronary artery disease and HF, that GDF15 is determined by age, hypertension, diabetes mellitus, coronary artery disease and HF, and that Troponin T is determined by age, sex, coronary artery disease and HF. In spite of, the determining effect of CHA_2_DS_2_-VASc, for all biomarkers, there are some small biomarker-specific effects.

**Table 2:** Overview of risk indicators per biomarker. EHRA = European heart rhythm association; LAVI = left atrial volume index; LVEF = left ventricular ejection fraction; AF = atrial fibrillation.

**NTproBNP**		**GDF15**		**Troponin T**
Age Sex EHRA category		Age Smoking LVEF (%)		Age Sex
CHA_2_DS_2_VASc - Age - Sex - Hypertension - Coronary artery disease - Chronic heart failure		CHA_2_DS_2_VASc - Age - Hypertension - Diabetes mellitus - Coronary artery disease - Chronic heart failure		CHA_2_DS_2_VASc - Age - Sex - Coronary artery disease - Chronic heart failure
Persistent AF				
LAVI (mL/m^2^)				
LVEF (%)				

In this multicenter, prospective study we showed that plasma levels of three biomarkers NTproBNP, Troponin-T and GDF-15 are higher in patients with persistent AF than in pAF. In those patients with higher biomarker levels, we observed higher co-morbidity burden and more unfavorable echo-parameters such as enlarged LAVI and slightly reduced LVEF. We furthermore observed that age and CHA_2_DS_2_-VASc (as surrogatefor higher comorbidity burden) are important determinants of all three biomarkers, and that progressive atrial disease, extrapolated from our findings of indicators of progressive atrial disease (higher EHRA category, increased LAVI, decreased LVEF and persistent AF) is a major determinant for plasma NTproBNP levels, but not for GDF15 or Troponin-T levels. Troponin-T and NTproBNP are both traditional cardiovascular biomarkers that are widely used in clinical practice, while GDF15 is a more novel cardiovascular biomarker. Troponin-T reflects myocardial damage and typically increases when ischemia is present or in case of coronary artery disease. Increased levels of troponin-T are associated with increased risk of all-cause mortality and major adverse cardiac events in patients with AF [6]. GDF-15 belongs to the transforming growth factor- β (TGF- β) cytokine superfamily [7] and circulating levels of GDF-15 are influenced by acute and chronic cellular stressors including ageing and disease. In healthy humans, GDF-15 levels are low throughout the entire body [8]. Increased plasma levels of GDF-15 are observed in various cardiovascular disease states such as HF and AF [9]. In this study we observed that plasma levels of Troponin-T and GDF-15 were not determined by persistent AF, but depended on other factors such as ageing, CHA_2_DS_2_-VASc, smoking, LVEF and sex. Therefore, these biomarkers may be useful to select those patients with higher co-morbidity burden and more unfavorable echo-measures, but not to assess more progressed AF disease states as persistent AF. NTproBNP, on the other hand, may reflect a biomarker that is helpful to identify those patients who are more likely to suffer from persistent AF than pAF. NTproBNP is a marker of myocardial stretch and is upregulated when additional stress is assessed on the left ventricle such as in HF or AF [10]. In patients with AF, higher levels of NTproBNP are associated with worse outcome including death, independent of CHA_2_DS_2_-VASc score [10]. Results of our study demonstrate that progression of AF is associated with increased NTproBNP levels, and it may therefore reflect an additional tool to select those patients with more persistent AF.

This study, however, is a cross-sectional study and results are thus descriptive and do not reflect causality of biomarker levels and AF burden. Because of its cross-sectional design this study cannot differentiate if increased biomarker levels are results of more persistent forms of AF or due to increased comorbidity burden. The fact that there is little biomarker-specific variance in comorbidities suggests that increasing plasma biomarker levels are not a sole effect of comorbidity burden, but future studies are warranted to evaluate this in more detail. Of note, AF-RISK was designed to evaluate AF-progression in patients with AF and did not include participants without AF; we could therefore not compare biomarkers levels in patients with and without AF. Not all AF patients were in an AF episode when blood withdrawal took place. This study was therefore unable to explore the effect of an AF episode on biomarker levels in pAF and persistent AF.

Overall, in this study we show that patients with persistent AF have higher plasma levels of NTproBNP, Troponin-T and GDF15, and that these higher plasma levels are accompanied by higher comorbidity burden and more unfavorable echocardiographic parameters. Additionally, we show that persistent AF determines NTproBNP levels, but not Troponin-T and GDF-15. We therefore suggest that NTproBNP is a better diagnostic biomarker than GDF15 and Troponin-T since NTproBNP is related to more AF, whilst GDF15 and Troponin-T are related to underlying conditions and not a specific AF phenotype. This study also suggest that in AF, Troponin T and GDF15, and especially NTproBNP could be used to detect those patients with high disease burden that may warrant more aggressive treatment of AF and concomitant comorbidities. Future studies, however, are essential to evaluate if more aggressive AF treatment and risk factor management will reduce disease progression and holds a novel therapeutic intervention to reduce the burden of AF.

## Funding

This work was supported by an unrestricted grant from Roche paid to the University Medical Center Groningen. AF RISK was supported by the Netherlands Heart Foundation (NHS2010B233).

**Trial Registration number**: Clinicaltrials.gov NCT01510210.

## Declaration of Competing Interest

The authors declare that they have no known competing financial interests or personal relationships that could have appeared to influence the work reported in this paper.
